# Clinicopathological Characteristics and Synchronous Lesions in Colorectal Cancer: A Single-Center Retrospective Colonoscopy Study

**DOI:** 10.3390/jcm14196715

**Published:** 2025-09-23

**Authors:** Vesna Brzački, Andrija Rančić, Snežana Tešić Rajković, Gordana Petrović, Ljubiša Rančić, Stanislava Mirković Dinić, Svetlana Jovanović

**Affiliations:** 1 Clinic for Gastroenterohepatology, University Clinical Center of Niš, 18000 Niš, Serbia; 2 Minimally Invasive Surgery Center, University Clinical Center of Niš, 18000 Niš, Serbia; 3 Clinic for Abdominal Surgery, University Clinical Center of Niš, 18000 Niš, Serbia; 4 Clinic for Endocrine Surgery, University Clinical Center of Niš, 18000 Niš, Serbia

**Keywords:** colorectal cancer, adenocarcinoma, colonoscopy, colorectal polyps, synchronous lesions, epidemiology, histopathology

## Abstract

**Background/Objectives:** Colorectal cancer (CRC) is a major global health concern, with rising incidence across age groups. Early detection via colonoscopy and identification of precancerous polyps are crucial for prevention and improved outcomes. The objectives were to evaluate the epidemiology, anatomical distribution, morphology, and histopathology of CRC, and its association with synchronous colorectal polyps. **Methods:** In 2023, a retrospective study was conducted on 1973 patients undergoing colonoscopy due to symptoms like blood in the stool, changes in bowel habits, abdominal pain, weight loss, anemia, or as CRC follow-up. Complete colonoscopies were performed, and suspicious lesions were biopsied or resected for histological evaluation. Statistical analysis was performed using SPSS 11.0. **Results:** CRC was diagnosed in 78 patients (3.95%), with a male predominance (70.51%, *p* < 0.05) and a mean age of 65.1 ± 8.9 years. The most affected age group was 61–70 years (43.58%). Tumors were most commonly located in the rectum (32.05%) and sigmoid colon (26.92%). Polypoid morphology was observed in 67.95% of cases. Adenocarcinoma was the predominant histological type (93.59%), followed by mucinous adenocarcinoma (6.41%), with significant differences between right and left colon (*p* < 0.001). Synchronous polyps were detected in 47.43% of CRC cases, primarily adenomas (60.22%). In 37.84%, the tumor and polyp were in the same colon segment. Men had a higher rate of synchronous polyps than women (*p* < 0.05). **Conclusions:** CRC is more common in older males and typically affects the rectosigmoid region. Adenocarcinoma is the leading type. Nearly half of patients had synchronous adenomas, highlighting the importance of full colonoscopy for early CRC detection and prevention.

## 1. Introduction

Colorectal cancer (CRC) is a major health concern due to its high and increasing incidence in most countries over the past three decades. Numerous risk factors, as well as protective factors, can significantly influence the development of this disease. CRC is the most common malignant tumor of the gastrointestinal tract and the second leading cause of death from cancer, after lung cancer. It ranks fourth in incidence among men and third among women. Improved screening and endoscopic removal of benign polyps, along with preventive measures, can help reduce its occurrence [1]. CRC predominantly affects the elderly, with 90% of cases diagnosed in patients older than 50. For individuals aged 80–85, the risk is 7–8 times higher than for those in the 50–55 age group. Nonetheless, CRC also occurs sporadically among younger individuals, with increased incidence reported in people under 40. Both sexes are equally affected, although some studies suggest a higher prevalence in men [2].

Although the exact cause of CRC is unknown, it is believed to result from a complex interaction of genetic and environmental factors. Common external risk factors include a diet high in meat and animal fats, physical inactivity, smoking, and alcohol use. About 70–90% of CRC cases develop from adenomatous polyps. Most CRCs originate from adenomas via the adenoma-carcinoma sequence, which typically spans 7–10 years. This indicates that the progression of CRC is generally slow, providing a window for polyp removal before cancer develops [3]. Two inherited conditions can predispose individuals to CRC: adenomatous polyposis syndromes and hereditary nonpolyposis colon cancer (HNPCC) [4]. Chronic inflammatory bowel diseases, especially ulcerative colitis and Crohn’s disease, increase CRC risk. It is about six times higher in ulcerative colitis and about three times in Crohn’s disease. Over 95% of CRCs are histologically adenocarcinomas, varying in cell maturity and mucin secretion [5,6].

The most common symptoms include blood in the stool, changes in bowel habits, abdominal pain, weight loss, anemia, and, rarely, perforation or ileus—signs indicative of a poor prognosis. However, symptoms can be less apparent, particularly when the tumor is located in the right colon. Most cancers are found in the rectum and sigmoid colon, followed by the cecum and ascending colon [7]. Key diagnostic procedures include digitorectal examination, rigid and flexible rectosigmoidoscopy, colonoscopy, and radiography. In daily practice, colonoscopy remains the “gold standard” for diagnosis, while endoscopic ultrasound and radiological methods are used to assess the extent of disease [8].

### Objectives of Research

The objectives of the research were to determine: the frequency of CRC by sex, the average age of patients with CRC, the frequency of CRC in specific age groups, external factors influencing the occurrence of CRC, the anatomical localization of CRC, the morphological and histological characteristics of CRC, the association of colorectal adenomas and CRC, the histological features of synchronous polyps, and the distance of polyps from CRC.

## 2. Materials and Methods

This retrospective study was conducted from January to December 2023 at the Clinic of Gastroenterohepatology, University Clinical Center Niš. It includes patients with symptoms such as blood in the stool, changes in bowel habits, abdominal pain, weight loss, and anemia, and those who have undergone colonic resection for CRC at least six months before 2023. The study did not include patients who underwent a partial colonoscopy and who suffered from hereditary bowel diseases. A total of 7 of our endoscopists had a high rating of cecum intubation (over 94%), with withdrawal time from 5 and a half to 8 min. For the colon examination, we used a video colonoscope from Olympus Corp., Hamburg, Germany. During the colonoscopic examination, the occurrence and localization of CRC, as well as its morphological characteristics, were recorded. The appearance of polyps associated with CRC and their distance from CRC were also documented. In this study, we did not use NBI and chromoendoscopy. The bowel preparation was also documented and calculated using the Boston bowel preparation scale (BBPS). We have included only patients with a BBPS higher than 7. Macroscopic lesions of the colon mucosa that were suspicious for CRC were biopsied 4 to 6 times with bioptic forceps, and the polyps were completely removed endoscopically (polypectomized). The biopsy samples were sent to the Institute of Pathology for histopathological analysis.

The collected data were analyzed using standard descriptive statistical methods such as mean, standard deviation, and percentage. The results were assessed using appropriate statistical tests. All data processing was performed using the Statistical Package for the Social Sciences (SPSS) software, version 11.0, in the Windows environment, with the results displayed in tables and graphs. Data are presented as arithmetic mean ± standard deviation, as well as minimum and maximum values for continuous variables, and as absolute and relative numbers for categorical variables. The incidence of colorectal cancer (CRC) by gender and age groups was compared using the *t*-test and one-way ANOVA, as appropriate. Associations between the presence of colon polyps and CRC, as well as the distance between CRC and colon polyps, were analyzed using the Chi-square test or Fisher’s exact test. A *p*-value of <0.05 was considered statistically significant.

## 3. Results

### 3.1. Incidence of CRC

During 2023 (January to December), colonoscopies were performed on a total of 1973 patients (1166 men and 807 women, with an average age of 64.7 ± 11.5) presenting with gastrointestinal symptoms or those who had undergone colon resection for CRC at least six months prior. CRC was confirmed endoscopically and verified by histopathological examination by a pathologist in 78 patients (3.95%). Of all 78 patients, there were 55 men (70.51%), with an average age of 65.1 ± 8.9; the youngest patient was 40 years old, and the oldest was 84. Also, there were 23 women (29.48%) with an average age of 62.8 ± 10.1 (Table 1). Statistically, CRC occurs more often in men than in women (*p* < 0.05).

CRC was diagnosed in 6 patients (7.69%) younger than 50 years; 16 patients (20.51%) aged 51 to 60 years; 34 patients (43.58%) aged 61 to 70 years; and 22 patients (28.20%) aged 71 to 80 years (Table 2). CRC occurred significantly more often in patients aged 61–70 years compared to those younger than 50 years (*p* < 0.05).

### 3.2. Anatomical Localization of the CRC

CRC was located in the rectum in 25 patients (32.05%), sigmoid colon in 21 (26.92%), descending colon in 13 (16.66%), transversum colon in 3 (3.85%), ascending colon in 10 (12.81%), and cecum in 6 patients (7.69%). A total of 6 (7.69%) of the patients, who had undergone surgery, had CRC distributed in the sigmoid colon (3 patients), rectum (2 patients), and transversum (1 patient). Synchronous CRC was detected in 1 patient (Table 3). Cancer of the rectosigmoid part of the colon was detected in 46 patients, occurring more frequently compared to cancers in other segments of the large intestine (*p* < 0.001).

### 3.3. Morphological Characteristics of CRC

The majority of CRCs had a polypoid appearance (67.95%). Fifteen patents with CRCs exhibited an annular appearance (19.23%), while 10 patients showed an ulcerative appearance (12.82%).

### 3.4. Histology Characteristics of CRC

Histological characteristics of adenocarcinoma were found in 73 patients with CRCs (93.59%), while mucinous adenocarcinoma was identified in 5 patients with CRCs (6.41%). The most common histological type in both parts of the colon was adenocarcinoma, with 67% of cases in the right colon and 89.33% in the left colon, and less common mucinous adenocarcinoma, accounting for 33% of cases in the right colon and 10.67% in the left colon. There are statistically significant differences in the distribution of certain histological types between the right and left colon (*p* < 0.001).

### 3.5. Association of the Appearance of Colorectal Polyps with CRC

#### 3.5.1. Incidence of Colorectal Polyps

Colorectal polyps were identified in 37 patients with CRC (47.43%). Colon polyps were not confirmed in 41 patients (52.56%). The majority of patients, 30 (81.08%), had one or two polyps, which is statistically significantly more common than in other groups with more than two polyps (*p* < 0.05). Three patients (3.84%) had more than 10 polyps (Table 4).

#### 3.5.2. Distance Between CRC and Colon Polyps

In 14 patients (37.84%), CRC and polyps were located in the same segment of the colon. In 7 patients (18.92%), the polyp was in the adjacent segment. In 9 patients (24.32%), the polyp was in a remote segment. One patient had polyps in both the same and adjacent segments. Three patients (8.11%) had polyps in the adjacent and remote segments. Another three patients (8.11%) had polyps in the same, adjacent, and distant segments (Table 5).

#### 3.5.3. Incidence of Colon Polyps in Specific Age Groups and Sexes

The ages of patients with colon polyps ranged from 40 to 78 years. Colon adenomas were diagnosed in 5 patients (13.51%) under the age of 50, and the highest number of patients with colorectal polyps were aged 61–70 (35.13%). The average age of patients with CRC without associated colon polyps was 65.9 ± 8.1, while the average age of those with CRC and associated colon polyps was 64.1 ± 9.8. There is no statistically significant age difference between the group with CRC and associated polyps and the group with CRC without associated polyps (*p* > 0.05).

Out of 37 patients with CRC who had synchronous colon polyps, 28 were men (35%) and 9 were women (53%). There is a statistically significantly higher occurrence of colon polyps and CRC in men compared to women (graph 9) (*p* < 0.05).

#### 3.5.4. Pathohistology of Colorectal Polyps

Out of the total number of polyps found, 78 (83.87%) were colon adenomas (tubular, tubulovillous, and villous). The other 15 (16.13%) were polyps with low risk of developing CRC, such as hyperplastic polyps.

## 4. Discussion

In Serbia, CRC is the second leading cause of death (after lung cancer) in the male population and the third (after breast and cervical cancer) in the female population. Based on mortality rates, Serbia is among the countries with high mortality. This high rate is due to the late detection of this cancer, resulting in weaker treatment outcomes. It also indicates a lack of adequate prevention. In Serbia, about two-thirds of patients are diagnosed at an advanced stage of the disease (stages III and IV), which significantly worsens treatment results.

Our research highlights the importance of colonoscopy as a diagnostic tool for evaluating patients with colon disease symptoms.

Age is one of the most important risk factors for the development of CRC. The incidence of CRC increases after the age of 40. CRC is a disease of the elderly, and over 90% of cases appear after the age of 50. Patients aged 60–79 years have a fifty times higher incidence of CRC than people younger than 40 years. In our study, the largest number of patients with CRC were in the 7th decade of life (43.58%). The frequency of CRC in the 8th decade of life was lower compared to the 7th decade, probably due to a smaller number of patients over 70 years of age who consult a gastroenterologist and undergo a colonoscopy. Unfortunately, CRC shows an increase in younger patients. In America, CRC is now one of the 10 most commonly diagnosed cancers in men and women aged 20–49 [9,10]. According to the data of Koketsu et al., the percentage of cancer in both the proximal and distal colon is higher in older patients than in younger patients [11].

CRC was diagnosed in 55 men (70.51%) and 23 women (29.48%). Statistically, CRC occurs more often in men than in women (*p* < 0.001). The frequency of CRC was twice as high in men then in women. Studies show a similar incidence of CRC in men and women, with a predominance of rectal cancer in men [12]. In the US, the incidence of CRC is 40% higher in men. Men tend to develop CRC slightly more often than women [13].

CRC was localized in the rectum in 25 patients (32.05%), in the sigmoid colon in 21 (26.92%), in the descending colon in 13 (16.66%), in the transversal colon in 3 (3.85%), and in the ascending colon in 10 (12.81%) patients. Newer epidemiological studies show that in developing countries, there has been a shifting of CRC from the sigmoid colon and rectum to the right side of the colon (cecum and ascending colon), which is primarily attributed to changes in eating habits, genetic factors, and preventive measures. The frequency of CRC in our series follows the results available in the literature [14]. No increase in the representation of CRC in the proximal part of the large intestine was observed, which can be explained by the earlier appearance of CRC symptomatology in the distal part of the large intestine [15].

The largest number of CRCs had a polypoid appearance (67.95%), 15 CRCs had an annular appearance (19.23%), and 10 CRCs had an ulcerative appearance (12.82%). The macroscopic appearance of CRCs varies according to their location, e.g., those of the proximal colon are predominantly larger exophytic changes that are often necrotic. In more distal parts of the large intestine, cancer more often involves the entire circumference of the intestine, causing an annular constriction of the lumen. We also found tumors that predominantly infiltrate the intestinal mucosa [16].

Histological characteristics of adenocarcinoma were found in 73 CRCs (93.59%), while mucinous carcinoma was found in 5 CRCs (6.41%). About 90% of CRCs are adenocarcinomas with glandular formations of different degrees of maturity and different mucin-secreting abilities. About 10% of colon cancers excessively secrete mucus, and those cancers where mucus makes up to 50% or more of the tumor mass are declared mucinous. Carcinomas of the scirrhous type make up only 1% of CRC [17]. In our study, the most common histological type was adenocarcinoma, with 67% of cases in the right colon and 89.33% in the left colon, and the rarest was mucinous adenocarcinoma, with 33% of cases in the right colon and 10.67% in the left colon. There are statistically significant differences in the representation of certain histological types of cancer between the right and left colon (*p* < 0.05). Pathohistologically, most CRCs are adenocarcinomas. The incidence of mucinous adenocarcinomas in Europe and the USA is about 10% [18], while in our study, 6.41% of cancers of this type were registered.

Colon adenocarcinoma, histologically, can be well, moderately, or poorly differentiated. This histological grade correlates with prognosis, but not as strongly as clinical stage, and it is not an independent prognostic indicator by itself [18]. Colorectal polyps were found in 37 patients with CRC (47.43%), most frequently localized in the rectosigmoid part. Colorectal polyps ranged in size from 1 mm to 20 mm. The largest number of patients with colorectal polyps were between 61 and 70 years old (35.13%). In terms of histological characteristics, the largest percentage was colorectal adenomas in 36 patients (92.3%) of patients. In terms of localization, CRC followed colorectal polyps, with the most frequent localization in the rectosigmoid part. In 14 patients (37, 84%), CRC and polyps are located in the same segment of the colon.

The incidence of colorectal adenomas (CRA) varies from country to country and follows the incidence of CRC. Previous studies found a positive correlation between the prevalence of CRA and CRC, with a correlation coefficient of 0.73 for men, 0.43 for women, and 0.67 for men and women combined [19,20,21]. These observations support the hypothesis that CRAs are an intermediate step in colon carcinogenesis. Our observations of an increase in the frequency of CRA with age are consistent with previous studies performed on autopsy material, where polyps occurred more often in persons over 60 years of age in countries with a Western lifestyle [22]. The development of carcinoma from an adenoma is represented as an adenoma-carcinoma sequence [3], which does not mean that all adenomas become carcinomas. Realistically, only 5% of all adenomas will progress to carcinomas [23]. There are two main histological types of polyps: hyperplastic and adenomatous. A hyperplastic polyp does not show dysplastic changes and is considered to have no malignant potential [24]. Risk factors for malignancy with hyperplastic polyps are: polyp size > 1 cm in diameter, localization in the right colon, and the presence of an adenomatous component in the polyp (mixed hyperplastic-adenomatous polyp), more than 20 hyperplastic polyps, family history of hyperplastic polyps, and family history of CRC. Adenomatous polyps show dysplastic changes, such as nuclear hyperchromatism, hyperplasia, or atypia [25,26]. The macroscopic appearance of polyps can be polypoid or non-polypoid. Polypoid can be peduncular or sessile. Non-polypoid lesions can be slightly elevated, completely flat, or slightly depressed [27,28]. The prevalence of nonpolypoid adenomas varies from study to study (8% to 42%), but is lower than polypoid adenomas. Progression to carcinoma is believed to be faster and more frequent in non-polypoid adenomas [27,29]. The risk of progression to cancer increases with adenoma growth ≥1 cm more significantly than with adenomas smaller than 1 cm [30]. Dysplasia means structural and cytological changes in the epithelial cell. These abnormalities are classified as low-grade or high-grade dysplasia, which subsequently increases the malignant potential [21]. In the literature, there is also the term “advanced adenoma” that describes an adenoma of ≥1 cm and/or with villous histology and/or high-grade dysplasia, with a high potential for progression to CRC [31].

The prevalence of colorectal polyps and CRC in our study was significantly higher in men than in women, which differs from the results of other studies in which the incidence of colorectal cancer was equal in both sexes [32]. The incidence of synchronous polyps is 36%, synchronous cancer 4.4% and metachronous cancer 3.5%. Synchronous CRC occurs more often with the presence of polyps [33]. In 11% of patients with definite synchronous polyps, synchronous CRC can be found. For patients with synchronous polyps, the incidence of metachronous CRC is 3.9% at 5 years and 6.5% at 10 years. The risk for recurrent polyps is 12% at 5 years, and is lower than in the general population with adenomas of the colon and rectum [34].

In the study by Borda et al., a high rate of CRC and synchronous polyps was noted. The high index of advanced adenomas and frequent synchronous lesions proximal to CRC stands out, which is why an incomplete colonoscopy can miss synchronous polyps and advanced adenomas in a high percentage [35]. Recent studies that analyzed the anatomical localization of CRC and colorectal adenomas indicated that there is no shift related to the localization of CRC, while the anatomical localization of colorectal adenomas shifts to the left colon in patients older than 70 years [36].

In our study, colorectal polyps were found in 37 patients with CRC (47.43%). The largest number of patients, 30 (81.08%), had one or two polyps, while 3 patients (8.11%) had more than 10 polyps. A total of 93 polyps were found. The largest number of patients with colorectal polyps is aged 61–70 (35.13%). They predominantly occur in patients over 60 years of age and are highly correlated with old age [35]. In elderly patients, due to the high incidence of synchronous tumors, a total colonoscopy should be insisted on. In patients with multiple adenomas, Schuman et al. found 66.7% of CRC in the cecum, so every colonoscopy in a patient with multiple adenomas must be performed up to the cecum [37]. There is a statistically more frequent occurrence of colorectal polyps and CRC in men compared to women. Synchronous tumors are most often localized in different segments of the colon, with the most common localization being in the sigmoid colon. The representation of synchronous tumors is approximately the same in adjacent and distant segments. When synchronous tumors are localized in one segment, sigmoid predominance is observed. Given the strong association with synchronous lesions, the detection of non-obstructive CRC in distal segments does not necessitate interruption of colonoscopy [38,39].

The role of flexible sigmoidoscopy becomes limited in the screening and diagnosis of CRC, given that the proximal half of the colon is not visualized endoscopically. One-third to one-half of the lesions are proximal to the sigmoid colon [40]. This ratio is even higher due to the movement of polyps and CRC towards the right half of the colon [41]. Sigmoidoscopy is inadequate for patients with distal CRC, considering that 3–5% with index CRC have synchronous cancers [42,43]. The presence of a proximal synchronous lesion affects the treatment of distal cancer. If the synchronous proximal lesion is malignant, the patient requires extensive bowel resection with removal of both lesions. If the proximal synchronous lesion is benign, it is possible to perform endoscopic polypectomy before surgical intervention. The finding of an adenomatous polyp or CRC during flexible rectosigmoidoscopy is an indication for colonoscopy.

The gold standard and the method of choice in the diagnosis of CRC is colonoscopy. The sensitivity of the method depends on the size of the pathological lesion located inside the colon. Thus, the sensitivity for polyps larger than about 1 cm is about 95%. Research conducted following individual patients has shown that with serial screening colonoscopies, the incidence of unseen lesions decreases with size and is about 5–6% for polyps larger than 1 cm [43,44]. In one of the largest randomized controlled trials conducted in the USA, a 20% reduction in the incidence of CRC was observed in a population that first underwent a test to detect occult bleeding from the colon, followed by colonoscopy and polypectomy if positive [45,46]. All the presented scientific facts support the conclusion that colonoscopy with polypectomy greatly reduces the incidence of CRC and, therefore, mortality.

## 5. Conclusions

This retrospective, single-center study highlights key clinicopathological characteristics of colorectal cancer (CRC), emphasizing its predominance in older male patients and its frequent localization in the rectosigmoid region. The majority of CRC cases were histologically classified as adenocarcinomas, with a significant proportion demonstrating polypoid morphology. Synchronous colorectal polyps were observed in nearly half of the CRC cases, predominantly in the same or adjacent segments of the colon, reinforcing the adenoma–carcinoma sequence as a central mechanism in colorectal carcinogenesis.

The findings underscore the crucial role of total colonoscopy not only in the early detection of CRC but also in the identification and removal of precursor lesions, particularly in patients over 60 years of age. Given the high prevalence of synchronous polyps and their potential for malignant transformation, comprehensive endoscopic evaluation should be standard in CRC diagnosis and surveillance strategies. These results support the continued use of colonoscopy as the gold standard in CRC screening and emphasize the importance of early intervention to reduce both incidence and mortality.

## Figures and Tables

**Table 1:** Incidence of CRC by gender and age.

Gender	N	Age	Min-Max
total	78 (100%)	65.1 ± 8.9	40.0–84.0
men	55 (70.51%)	66.0 ± 8.4	45.0–79.0
women	23 (29.48%)	62.8 ± 10.1	40.0–84.0

**Table 2:** Incidence of CRC by age groups.

Incidence of CRC	
Age (God)	n
<50	6 (7.69%)
51–60	16 (20.51%)
61–70	34 (43.58%)
>70	22 (28.20%)
**Total:**	78

**Table 3:** CRC distribution by anatomical localization.

Anatomical Localization	n	(%)
Rectum	25	32.05
Sigma	21	26.92
Descendens	13	16.66
Cecum	6	7.69
Ascendens	4	5.12
Transversum	3	3.85
**Operated**	6	7.69
Sigma	3	
Transversum	1	
Rectum	2	
Total:	78	100%
1 patient: rectum-cecum		

**Table 4:** Association of the appearance of colon polyps with CRC.

Number of Polyps		
0		41 (52.56%)
1	16 (20.51%)	37 (47.43%)
2	14 (17.95%)	
3	2 (2.56%)	
5	1 (1.28%)	
8	1 (1.28%)	
10	3 (3.84%)	
**Total:**	37	

**Table 5:** Distance between CRC and colon polyps.

Proximity to Polyps	n	(%)
Same segment	14	37.84
Nearby segment	7	18.92
Remote segment	9	24.32
Same and nearby segment	1	2.70
Nearby and remote segments	3	8.11
Same, nearby, and remote segments	3	8.11
**Total:**	37	100%

## Data Availability

The data presented in this study are available on request from the corresponding author.
